# Key-interventions derived from three evidence based guidelines for management and follow-up of patients with HFE haemochromatosis

**DOI:** 10.1186/s12913-016-1835-2

**Published:** 2016-10-13

**Authors:** Annick Vanclooster, Hub Wollersheim, Kris Vanhaecht, Dorine Swinkels, Bert Aertgeerts, David Cassiman, on behalf of the Haemochromatosis working group, Rene Westhovens, Johan Van Cleemput, Vincent Maertens, Hans Van Vlierberghe, Rudy Harlet, Patrick Verschueren, Walter Droogne, Koen Theunissen, Neree Claes, Hannelore Van Droogenbroek, Patrik Vankrunkelsven, Chantal Mathieu, Sabien Severi, Ilse Scherens, Sandra Rottiers

**Affiliations:** 1 Department of Hepatology and Metabolic Center, University Hospital Gasthuisberg, Herestraat 49, 3000 Leuven, Belgium; 2 Scientific Institute for Quality of Healthcare, Nijmegen Centre for Evidence Based Practice, Radboud University Medical Centre, Nijmegen, The Netherlands; 3 Department of Public Health and Primary Care, Health Services Research Group, KU Leuven, Leuven, Belgium; 4 Department of Laboratory Medicine, Laboratory of Genetic Endocrine and Metabolic diseases, Radboud University Medical Centre, Nijmegen, The Netherlands; 5 Academic Center for General Practice, KU Leuven, Leuven, Belgium

**Keywords:** Hereditary haemochromatosis, Key-interventions, Recommendations, Consensus

## Abstract

**Background:**

HFE-related hereditary haemochromatosis (HH) is a common autosomal recessive disorder with clinical manifestations ranging from asymptomatic disease to possible life-threatening complications. Cirrhosis, hepatocellular carcinoma, diabetes mellitus or osteoporosis can develop in HH patients not treated or monitored optimally. The purpose of this study was to develop key-interventions (KI’s) to measure and improve the quality of care delivered to patients diagnosed with HH.

**Methods:**

A RAND-Modified Delphi method was used to develop KI’s. In the first round of a scoring form to prioritize the recommendations extracted from evidence-based guidelines was circulated between experts. The results of this survey were discussed in a consensus meeting, followed by a final appraisal of the selected recommendations. This resulted in a list of measurable KI’s.

**Results:**

Initially, 41 key recommendations on screening, diagnosis and treatment/management were extracted from three existing guidelines on HH (European Association for the Study of the Liver, American Association for the Study of Liver Diseases and Dutch guideline on HH). Finally, a core set of 24 recommendations resulted in 15 KI’s.

**Conclusions:**

This manuscript presents the results of the process to develop KI’s to measure and improve the quality of care for patients with HH.

**Electronic supplementary material:**

The online version of this article (doi:10.1186/s12913-016-1835-2) contains supplementary material, which is available to authorized users.

## Background

HFE-related hereditary haemochromatosis (HH) is a common chronic autosomal recessive disorder, with a genetic prevalence of 1/200 to 1/400. It has an estimated carrier frequency of 1/10 in those from Northern European descent. The phenotype results from inappropriate accumulation of iron, resulting in end-organ damage [1]. Symptoms can be absent, but complications may also be debilitating or even fatal such as diabetes mellitus, osteoporosis, cirrhosis or hepatocellular carcinoma [2–4]. Patients with HH are seen, due to the variety of symptoms, by many different professionals, ranging from hepatogastroenterologists, hematologists, rheumatologists, general practitioners to nurses. The varying published criteria for case definition, referral, diagnosis, interpretation of test results, follow-up and family screening approaches may lead to confusion in the diagnosis, treatment and follow-up process for physicians, patients and their relatives. Almost 20 years after the description of the causal gene defect in the HFE gene, allowing the definite diagnosis of HH, the level of understanding of the medical risk associated with adequate vs. inadequate treatment and follow-up, with regard to the disease-associated complications described above, is virtually unchanged. For instance, there is no clear view on the risk of developing diabetes, once treatment is installed [5], compared to the risk in the general population, let alone the risk of developing osteoporosis or hepatocellular carcinoma. One of the factors hampering progress in our understanding of the disease-associated risk, in our opinion, is the lack of well-defined standards (i.e. key-interventions, KI’s) for screening, treatment and follow-up of HH. International guidelines on HH exist [6–9] but, as we demonstrated recently, their applicability is limited as they fail to describe how long-term follow-up should be organized and evaluated [10].

To enable treatment evaluation, to support improvement of delivered care and to assist professionals with the delivery of optimal care to their HH patients, we developed KI’s for HH. This study is unique, as the KI’s are formulated, starting from recommendations based on the integration of three evidence-based guidelines.

## Methods

**Fig. 1:**
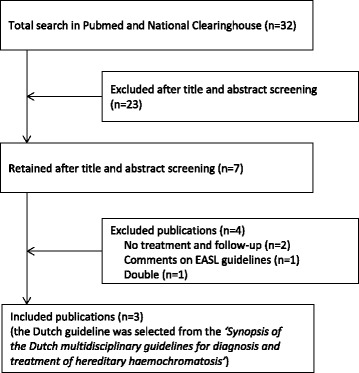
Selection of guidelines

A RAND-modified Delphi method was used to develop key-interventions (KIs) related to screening, diagnosis, treatment and follow-up of patients with HH in five steps, see Fig. 1 [11, 12]. This method combines evidence-based practice with expert opinion by using a multidisciplinary panel in the systematic process of developing KIs [13].

### Extraction of recommendations

References to guidelines on HH were searched in the Medline database as well as the National Guideline Clearinghouse using following MESH terms: ‘haemochromatosis’ and ‘practice guideline (publication type)’ or ‘practice guidelines as topic’ (June 2013). Only evidence-based guidelines with clearly defined recommendations published over the last 10 years were included. If the guideline had been updated, the latest version was used. We included three guidelines from different professional organizations: European Association for the Study of the Liver (EASL), American Association for the Study of Liver Diseases (AASLD) and Netherlands Association of Internal Medicine (NIV), the Netherlands Society of Clinical Chemistry and Laboratory Medicine (NVKC) and Association of Laboratory Physicians (VAL) (DUTCH). These three guidelines on screening, diagnosis and treatment/management of patients with primary HH were selected because of their clearly defined recommendations [6–10]. The selected guidelines describe evidenced-based recommendations, whether or not in combination with expert opinion recommendations [10]. One researcher (AV) extracted the recommendations from the three guidelines. All recommendations were classified into either ‘screening’, ‘diagnosis’ or ‘treatment/management’, together with their Level of Evidence (LoE), where possible. If a recommendation had a slightly different phrasing or if contradictory messages in different guidelines were encountered, this was mentioned in ‘comments’ (see Additional file 1: Appendix A).

To evaluate these recommendations, a written scoring form was sent to a multidisciplinary team of experts who are involved in the routine care of patients with HH. This multidisciplinary team (*n* = 17) consisted of three hepatogastroenterologists, two rheumatologists, two cardiologists, two endocrinologists, two hematologists, three general practitioners and three nurses.

### Written scoring form

Forty-one recommendations were sent to the multidisciplinary expert team by e-mail (see Additional file 1: Appendix A). They were asked to score the recommendations on a nine point scale taking into account the following question: *‘Is performing this recommendation important for the delivery of high quality of care for patients with HH?’* (one point for a bad measure (i.e. no benefit for the patient) and up to nine points for an excellent measure). The multidisciplinary team was asked to take into account ‘health gain’ (morbidity, mortality, quality of life), ‘patient burden’ and ‘side effects’ when scoring the recommendations. When it was impossible for a team member to judge the statement, he/she could mark it as ‘impossible to judge’. Panel members were encouraged to add new recommendations or make changes to the existing recommendations. Subsequently, the team members were asked to prioritize the three most relevant recommendations for each domain (screening, diagnosis and treatment/management). The scoring form was filled out by 15 of 17 experts (see Additional file 2: Appendix B). One endocrinologist did not respond despite several reminders. One endocrinologist only suggested two extra recommendations regarding diabetes mellitus and did not score the proposed recommendations.

A summary report was drafted, based on the individual results of the written scoring form to facilitate the consensus meeting. This summary report marked the recommendations as having received a ‘low’, ‘uncertain’ or ‘high’ potential to deliver good quality of care in patients with HH from the experts. Three selection criteria were taken into account, i.e. pre-selection, top-3 percentage and agreement between scorers, which led to a final selection of recommendations.

Pre-selection was summarized into an overall ranking score and median rating. Each participant had to score the top three for screening, diagnosis and treatment/management. Calculation of this overall rating existed of ranking a recommendation first, second or third with three, two and one point(s) respectively. Taking into account the maximum score (number of panel members who scored this item multiplied by three), the result was converted into percentages. This methodology is described in reference [13].

High potential recommendations were those with a high overall ranking score (top-3 percentage > 20 %) as well as a high median score (≥8). If a recommendation had both a low score on overall ranking (overall ranking of 1–20 % or < 1 %) and median rating (<8), this recommendation was classified as a recommendation with low potential. Other combinations were classified as recommendations with an uncertain potential (median score < 8 and top-3 percentage > 20 %; overall ranking of 1–20 % and median score of ≥ 8) (Table 1). A cutoff for overall rating of 20 % and a median score of ≥ 8 was associated with good reproducibility and reliability as well as face validity [13].

**Table 1:** Key recommendation classification into categories of high, uncertain and low potential according to their overall ranking score and median score

Overall ranking score	Median score ≥ 8	Median score < 8
Top-3 percentage > 20 %	High potential (+)	Uncertain (+/-)
1–20 %	Uncertain (+/-)	Low potential (-)
<1 %	Low potential (-)	Low potential (-)

### Criteria for (dis)agreement

The evaluation of agreement between the members of the expert panel showed agreement in scoring by a ≥ 70 % scoring in the highest tertile (7–9) (Table 2A). If ≥ 30 % of the panel members scored in the lowest tertile (1–3) and ≥ 30 % scored in the highest tertile (7–9) (Table 2B), there was disagreement. Such an item became a discussion point for the consensus meeting. All other combinations resulted in no selection (Table 2C).

**Table 2:**
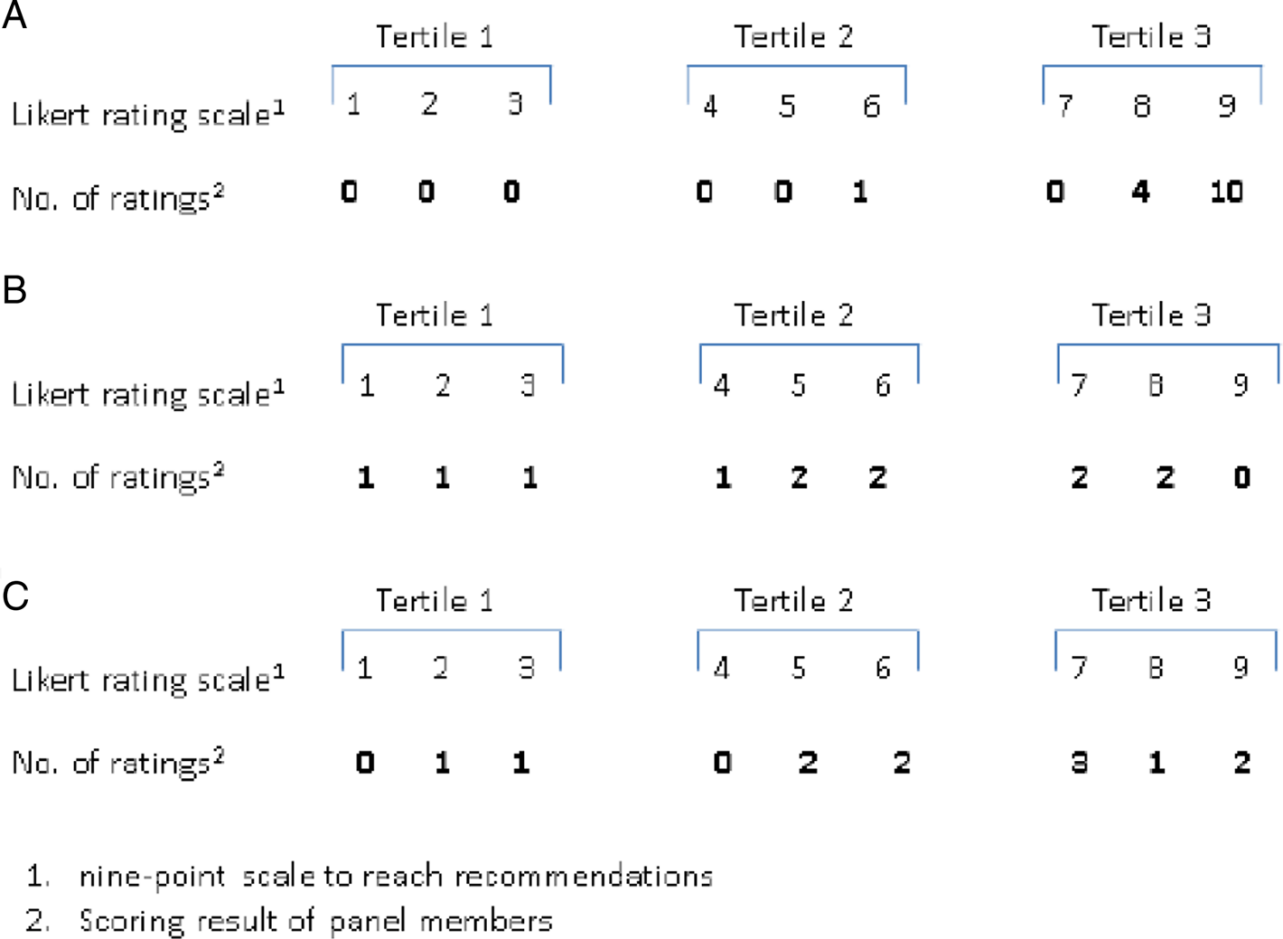
Examples of agreement and disagreement between panel members

A: Agreement (+: selection), B: Discussion Point (+/-), C: Disagreement (–: no selection)

The items selected for the consensus meeting, were those with a high potential if pre-selection and agreement were positive. A low potential was given to those recommendations which were not pre-selected and showed no agreement or those without agreement and a questionable pre-selection (no selection). All other combinations resulted into recommendations with an uncertain potential (discussion) with regard to delivering high quality of care for patients with HH (Table 3).

**Table 3:** Selection of items after scoring

Preselection	Agreement	Final label
+	–	Discussion
–	–	No selection
–	+	Discussion
+	+	Selection
+/-	+	Discussion
+/-	–	No selection
+/-	+/-	Discussion
+	+/-	Discussion
–	+/-	No selection

### Consensus meeting

A face-to-face consensus meeting was organized to discuss the recommendations. Consensus was agreed between a hepatogastroentrologist, a hematologist, a cardiologist, a rheumatologist and a specialized nurse. It took 90 min to discuss the 41 recommendations. An overview of the ‘high’, ‘low’ and ‘uncertain’ potential of each recommendation was given to the panel members (see Additional file 3: Appendix C). Three additional recommendations completed the list for the panel. Recommendations with a high potential were included. There was discussion about the items with an uncertain potential and the extra recommendations for inclusion or rejection. There was also discussion about rejection in case of ‘low potential’ recommendations. This discussion was moderated by an independent researcher (DS) who did not participate in the scoring of the recommendations.

### Final appraisal of recommendations

The final list of selected recommendations was sent to all scoring panel members after the consensus meeting (see Additional file 4: Appendix D). All 15 panel members agreed with the core set of recommendations.

### Development of key-interventions

The recommendations were subsequently translated into key-interventions for patients with an established diagnosis of HH. These key-interventions were formulated by two researchers (AV and DC).

## Results

Three guidelines met our search criteria: 1 European (EASL), 1 American (AASLD) and 1 guideline from the Netherlands (Dutch) (Fig. 1) [6–9].

**Fig. 2:**
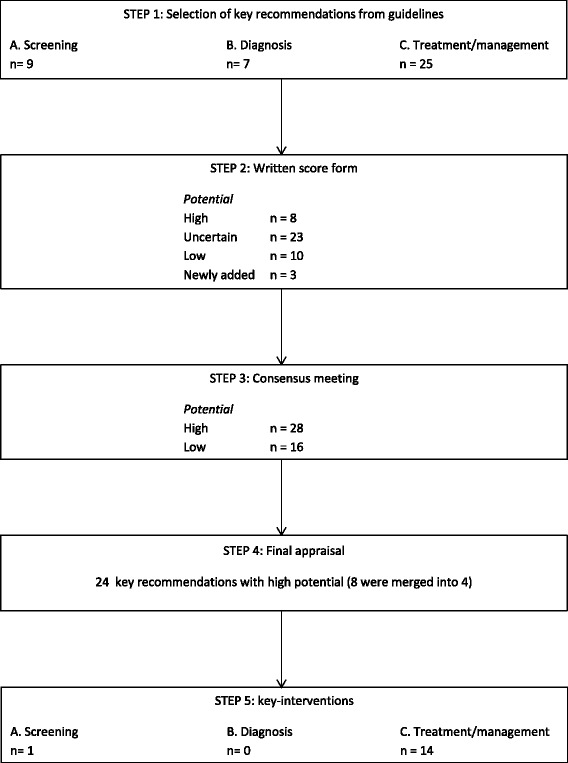
Selection of key-interventions

At the start, 41 recommendations were extracted from the three guidelines (see Additional file 1: Appendix A). Key recommendations were assigned to three domains: screening (9), diagnosis (7) and treatment/management (25) (Fig. 2). Where possible, the level of evidence was reported (see Additional file 1: Appendix A).

Pre-selection (by priority and median score) and agreement resulted in the inclusion of 28 and exclusion of 13 recommendations, after the consensus meeting. The three suggested additional recommendations were also excluded: two were related to screening of HH in patients with diabetes mellitus, the third was related to age restrictions to start phlebotomies. The panel decided that a general recommendation about age restriction could not be given, but that on the contrary there was a need for an evaluation of each individual case. From the total of 28 included recommendations, eight were merged into four recommendations as their content overlapped. Finally, 15 of the 24 recommendations were transformed into KI’s (Table 4). Nine initial recommendations were excluded because they could not be transformed into measurable indicators or were related to screening for HH, while our study aims at patients with an established diagnosis of HH. For instance: *‘HFE testing should be considered in patients with type 1 diabetes in case of abnormal iron parameters’* (Table 5).

**Table 4:** Key-interventions to treat patients with HH

Screening
1. First-degree relatives of HH patients must be screened.
Treatment/management
Phlebotomy
2. HH patients with raised ferritin levels must start treatment with (bi)weekly phlebotomy (removing 400–500 ml of blood). 3. The ferritin target level for HH patients ‘on treatment’ is between 50–100 μg/L. 4. HH patients without indicators of significant liver disease (AST and/or ALT elevation), but with elevated ferritin, must also proceed to phlebotomies. 5. HH patients with advanced liver fibrosis or cirrhosis can safely undergo phlebotomy and must also be treated. 6. HH patients undergoing phlebotomies must be advised to take adequate hydration before and after treatment and avoid vigorous physical activity for 24 h after treatment.
Examinations
7. Screening for liver fibrosis or cirrhosis in HH patients must be performed and can be performed using either transient elastography or biopsy.
General issues
8. HH patients without evidence of iron overload must be monitored annually and treated when the ferritin rises above normal. 9. HH patients must be immunized against hepatitis A and B. 10. HH patients with cirrhosis must receive yearly influenza and 5-yearly pneumococci vaccination. 11. HH patients with cirrhosis must be screened every 6 months for focal liver lesions (by ultrasound and serum alpha fetoprotein). 12. HH patients must be assessed and managed for complications (liver disease, diabetes mellitus, joint diseases, endocrine deficiency (hypothyroidism), cardiac disease, porphyria cutanea tarda and osteoporosis). 13. Fasting glycemia and/or HbA1c must be measured yearly in HH patients. 14. HH patients who have complaints compatible with osteoarthritis, must undergo physical and radiological evaluation.
Diet/lifestyle 15. HH patients in the iron depletion phase must avoid the intake of alcohol.

**Table 5:** Recommendations to screen for HH in other patient populations

Screening
1. HFE testing must be considered in patients with well-defined chrondrocalcinosis in case of an otherwise unexplained increase in ferritin and transferrin saturation. 2. HFE testing must be considered in patients with type 1 or type 2 diabetes in case of abnormal iron parameters. 3. HFE testing should not be performed in patients with unexplained osteoarthritis. 4. HFE testing should be considered in patients with unexplained chronic liver disease pre-selected for increased transferrin saturation.
Diagnosis
5. Patients from liver clinics should be screened for transferrin saturation and serum ferritin. 6. Patients from liver clinics should be offered genetic testing if transferrin saturation and ferritin are increased. 7. If a patient has suggestive symptoms, physical findings or a suggestive family history, a combination of TS and ferritin should be measured. If both are abnormal (TS > 45 % and ferritin above upper limit of normal), HFE mutation analysis should be performed. 8. HFE testing for the C282Y and H63D polymorphism should be carried out in all patients with otherwise unexplained increased serum ferritin and transferrin saturation. 9. In C282Y homozygote patients with increased iron stores, liver biopsy is no longer necessary to diagnose haemochromatosis.

### Screening

‘*First-degree relatives of HH patients must be screened’* is highlighted as the most important recommendation in the ‘screening’ section and the only one which could be transferred into a KI. All three guidelines (EASL, AASLD and Dutch) promote screening of first-degree relatives in patients with HH [6–8]. Other recommendations were excluded (i.e. screening of patient populations, presenting with e.g. chronic hepatitis or other symptoms possibly related to HH) since they were not related to HH patients and are therefore outside the scope of our study.

### Treatment/management

The treatment and management section was subdivided into ‘phlebotomy’, ‘examinations’, ‘general issues’ and ‘diet/lifestyle’.

#### Phlebotomy

All recommendations regarding phlebotomy were transferred into KI’s. Two of them were merged into one KI. There was consensus between the three guidelines, i.e. ‘*HH patients with raised ferritin levels must start treatment with (bi)weekly phlebotomy (removing 400–500 ml of blood)’*. The AASLD clearly promotes a target level of serum ferritin (SF) between 50 and 100 μg/L [7]. On the other hand, the Dutch guideline requires a target SF level under the upper limit of normal [8]. As important differences between the different laboratories reporting transferrin saturation (TS) and SF occur, the expert panel agreed with targeting a SF level between 50 and 100 μg/L, to have a clear cut-off point. There was consensus between the three guidelines as well as between the experts, that patients with organ damage should undergo phlebotomies in case of iron overload.

#### Examinations

The EASL guideline describes that patients must undergo examinations (transient elastography (*(fibroscan), a non-invasive liver stiffness measurement (Echosens, France))*, liver biopsy) in order to detect liver damage early [6]. During the consensus meeting, two recommendations were merged into one, which was transformed into the following KI *’Screening for liver fibrosis or cirrhosis in HH patients must be performed and can be performed using either transient elastography or biopsy’.*

#### General issues

In contrast to the Dutch guideline, which requires monitoring C282Y homozygotes without iron overload every 3 years, the EASL as well as the AASLD require annual monitoring [6–8]. Considering that annual monitoring is easier to organize for the health care providers, the expert panel decided that patients should monitor their SF yearly (although this is more costly) and start treatment when the ferritin level rises above the upper limit of the reference range.

Immunization against hepatitis A (HAV) and B (HBV)is an important issue in patients with HH because patients with a chronic liver disease are at higher risk to develop complications of HAV and HBV [14]. In addition, patients with cirrhosis must be immunized against influenza yearly and pneumococci every 5 years. All three recommendations are supported by the EASL and were transformed into a KI.

During the consensus meeting, the recommendation to screen cirrhotic patients for focal liver lesions by ultrasound as well as measurement of serum alpha fetoprotein every 6 months, was not challenged and supported by the European as well as the Dutch guidelines [6–8].

Patients with HH can develop a variety of medical problems, including liver disease, diabetes mellitus, joint disease, endocrine deficiencies (hypothyroidism, hypogonadism), cardiac disease, porphyria cutanea tarda and osteoporosis [2–4]. Assessment and eventual management of these problems are necessary in order to prevent patients developing worse or additional complications. Considering this, the expert panel agreed that patients should have their fasting glycemia and/or HbA1c checked yearly, to detect diabetes mellitus early. Furthermore, the following EASL recommendation: *‘physical and radiological evaluation is necessary to evaluate possible arthralgia and arthritis’* was changed during the consensus meeting into *‘HH patients who have complaints compatible with osteoarthritis, must undergo physical and radiological evaluation’.* All these recommendations were transformed into KI’s (Table 4).

#### Diet/lifestyle

In theory, additional iron taken up via the diet or via supplements can be removed via phlebotomy. In general, however, most physicians will advise their patients to avoid extra iron uptake from the diet, by avoiding Vitamin C supplements taken with meals, as well as the intake of iron supplements. The expert panel considered that every person, whatever his/her health status is, needs a healthy diet. Therefore we only selected the recommendation that patients at start of treatment must be advised to avoid the intake of alcohol.

### Recommendations that were not selected

Nine out of 24 recommendations which were highlighted as recommendations to deliver good quality of care were not selected to be transformed into KI’s. Four were derived from the screening section of the recommendations. Secondly, five diagnostic recommendations could not be transformed in quantifiable KI’s (Table 5). The nine recommendations that did not qualify as KI’s, were all valid recommendations but not related to the measurement of the quality of treatment or management of patients who are already diagnosed with HH.

## Discussion

This is the first study describing the development of 15 guideline-based KI’s, derived from three evidence-based guidelines on HH, by a multidisciplinary expert panel. The KI’s relate to screening, diagnosis and treatment/management of HH. The final list of 15 KI’s provides professionals with parameters to measure and follow-up quality of care in patients with HH, thereby preventing the development of serious, potentially fatal complications in this patient population.

The uniqueness of this study lies in the fact that we developed KI’s by starting from three evidence-based guidelines, and followed by applying a RAND modified Delphi method to develop consensus. Evidence-based KI’s are the active ingredients in care pathways (CPs) [15, 16]. CPs aim to improve patient processes and outcomes by (re)organizing care processes. The European Pathway Association defines a CP as “a complex intervention for the mutual decision making and organization of care for a well-defined group of patients during a well-defined period” [17]. The integration of CPs in a well-defined group of patients was already described in several patient settings, i.e. Chronic Obstructive Pulmonary Disease, Proximal Femur Fracture, patients with stroke [18–20]. This resulted in better inter-professional teamwork, a higher level of organized care and a lower risk of burnout in the case of CPs in acute health care teams [19]. By using CPs, the care delivered to stroke patients resulted in more effective treatment and better use of organized care [20]. Clinical content of an evidence-based CP can be created through the eight-step method developed by Lodewijckx et al. [18]. Our selected KI’s will indeed be used to create a CP by using hospital-related indicators,and including patient and organizational indicators will also be considered.

Earlier research shows that outcomes are influenced by the composition of the expert panel [21]. The strength of our study is that 15 independent clinical experts scored the 41 recommendations. The experts were selected from six different hospitals (tertiary university hospitals, as well as regional hospitals), from primary care and from two universities in Belgium. The appraisers are active in 7 different medical specialties (see Additional file 2: Appendix B). The consensus meeting was headed by an independent expert (DS) in HH, from the Netherlands. A limiting factor of our study is that only 5 experts participated in the consensus meeting, for logistical reasons and that no patient representatives were involved. Nevertheless, only the opinion of a general practitioner was absent from the consensus meeting and all 15 appraisers agreed with the final list of 15 recommendations, drafted after the consensus meeting.

The resulting set of KI’s, which is a combination of evidence and expert opinion, is developed to be applicable in different health care settings: hospitals as well as primary care settings. For this reason, the set of KI’s can be used by different health care professionals, i.e. physicians (hepatogastroenterologists, hematologists, rheumatologists, general practitioners, …) as well as advanced nurse practitioners [22].

Although we did not probe for prioritization between the recommendations, at the consensus meeting, the most important recommendation is clearly that “patients with raised ferritin levels should start treatment with (bi) weekly phlebotomy” (recommendation nr. 2), which is also highlighted in the questionnaire round (see Additional file 3: Appendix C) and by the three guidelines [6–8]. No recommendation about apheresis in HH patients is included, since this was not described in the referring guidelines. The goal of treatment is to prevent HH patients from developing complications of the disease and, therefore, patients should be assessed for complications and those need to be managed in case they are present. A longitudinal follow-up, applying the KIs we propose here, may in addition finally reveal whether patients with HH – treated adequately – still have an increased risk to develop diabetes.

As described in Additional file 1: Appendix A, there is a difference in wording of the recommendations between the three selected guidelines, but there are no important contradictions. During the consensus meeting, some recommendations were merged or adapted in view of the development of KI’s (Additional file 4: Appendix D).

Limitations to this study are that patient representatives are not included in the expert panel notwithstanding the fact that HH patients in general are able to assume control of their own care process. We are therefore planning further qualitative research on how patients experience the quality of the delivered care by face-to-face interviews, questionnaires and focus group interviews. We are particularly interested in further care coordination in primary care and self-management by patients, since integrated patient care is becoming more and more important [23].

The final list of 15 recommendations forms the basis for the measurement of the quality of the usual clinical care and allows to objectively quantify the effects of adaptations to the care path of the HH patient population. A practice test to assess the measurability of the KI’s is planned, as well as a study demonstrating improvement of outcome, when the KI’s are applied.

## Conclusion

HH is a common disorder with potentially life-threatening complications, if left untreated or not treated properly before irreversible organ damage occurs. To optimize the care for HH patients, we developed a set of 15 measurable clinical KI’s. This set of standardized KI’s now allows us to measure, adapt and deliver high quality of care for patients with HH in the hospital setting as well as in primary care.

## Additional files

Additional file 1: Appendix A. Selection of recommendations. (DOCX 209 kb)
Additional file 2: Appendix B. Expert overview. (DOCX 14 kb)
Additional file 3: Appendix C. Overview scores written questionnaire. (DOCX 64 kb)
Additional file 4: Appendix D. Selected recommendations. (DOCX 18 kb)

## Abbreviations

AASLD: American Association for the Study of Liver Diseases
CP: Care Pathway
DUTCH: Dutch guidelines for hereditary haemochromatosis
EASL: European Association for the Study of the Liver
HAV: Hepatitis A virus
HBV: Hepatitis B virus
HH: Hereditary haemochromatosis
KI: Key-intervention
LoE: Level of Evidence
SF: Serum ferritin
TS: Transferrin saturation
