# Supplementary material for: Key-interventions derived from three evidence based guidelines for management and follow-up of patients with HFE haemochromatosis
Source: BMC Health Serv Res. 2016 Oct 13;16:573. doi: 10.1186/s12913-016-1835-2 (PMC5062877; doi:10.1186/s12913-016-1835-2)
Supplement: Additional file 1: Appendix A. — Selection of recommendations. (DOCX 209 kb) [file 12913_2016_1835_MOESM1_ESM.docx]

**Questionnaire recommendations Hereditary Haemochromatosis**

1. **Instructions**
2. **Questionnaire**
3. **Attachment I-III**
   1. **Process of indicator development**
   2. **References**
   3. **Level of Evidence**
4. **Instructions**
   1. **Score**

The recommendations, extracted from different international guidelines, have to be scored on a 9-point likert scale by the expert panel you are attending, taking into account the following questions: ‘Is performing this recommendation important for the delivery of high quality care for patients with hereditary haemochromatosis?’, with score ‘1’ representing a very bad measure and score ‘9’ an excellent measure. When it is impossible for you to judge the statement, you can mark it as well. In order to judge a recommendation, it’s important to take into account **health gain** (morbidity, mortality, quality of life), **patient burden** and **side-effects. Please pay attention:** the statements can either be described as a positive or a negative sense.

Help to your choice: The level of evidence and corresponding guideline(s) are marked for each recommendation, if possible. ‘TEXT’ is filled in if the recommendation is extracted from the text of the guidelines. Information from other guidelines, not fully supporting the aforementioned recommendation is reported under ‘comments’.

Note: In addition to the guidelines, one extra article for haemochromatosis is referred to as well.

- 1. **Prioritization**

For screening, diagnosis, management and follow-up, you are asked to select and prioritize the three most relevant recommendations in order to measure high quality of care for Hemochromatosis patients.

- 1. **Time schedule**

28/10/2013: Recommendations to appraisers
20/11/2013: Final date to send in the scoring
19/12/2013: **Consensus meeting: 15.00h in Bach, administrative building**

- 1. **Additional recommendations/modifications**

If you don’t fully agree to this set of recommendations, you can add supplemental items and modify the proposed ones. It’s also possible to add recommendations explicitly **discouraging** a particular procedure.

NOTE:

Attachment I: Process of indicator development

Attachment II: Reference articles / guidelines

Attachment III: Level of evidence for the corresponding guidelines

**2. Questionnaire**

| **1. SCREENING** | | | | | |
| --- | --- | --- | --- | --- | --- |
| **Performing this recommendation is important for the delivery of high quality care for patients with hereditary haemochromatosis** | | | | | |
|  | **Recommendation** | **LoE** | **Gdln** | **Comments** | **Score** |
| 1 | Population screening should **NOT** be done | 1B | EASL  AASLD |  | **1 2 3 4 5 6 7 8 9**  **<----------------------------------------------------------->**  **disagree agree**  Impossible to judge □ |
| 2 | Genetic testing of first-degree relatives should be considered | 1B  1A  2 | EASL  AASLD  DUTCH |  | **1 2 3 4 5 6 7 8 9**  **<----------------------------------------------------------->**  **disagree agree**  Impossible to judge □ |
| 3 | HFE testing must be considered in patients with porphyria cutanea tarda | 1B | EASL |  | **1 2 3 4 5 6 7 8 9**  **<----------------------------------------------------------->**  **disagree agree**  Impossible to judge □ |
| 4 | HFE testing must be considered in patients with well-defined chondrocalcinosis | 2C | EASL |  | **1 2 3 4 5 6 7 8 9**  **<----------------------------------------------------------->**  **disagree agree**  Impossible to judge □ |
| 5 | HFE testing must be considered in patients with hepatocellular carcinoma | 2C | EASL |  | **1 2 3 4 5 6 7 8 9**  **<----------------------------------------------------------->**  **disagree agree**  Impossible to judge □ |
| 6 | HFE testing must be considered in patients with type 1 diabetes | 2C | EASL |  | **1 2 3 4 5 6 7 8 9**  **<----------------------------------------------------------->**  **disagree agree**  Impossible to judge □ |
| 7 | HFE testing should **NOT** be done in patients with unexplained arthritis or arthralgia | 1C | EASL |  | **1 2 3 4 5 6 7 8 9**  **<----------------------------------------------------------->**  **disagree agree**  Impossible to judge □ |
| 8 | HFE testing should **NOT** be done in patients with type 2 diabetes | 1B | EASL |  | **1 2 3 4 5 6 7 8 9**  **<----------------------------------------------------------->**  **disagree agree**  Impossible to judge □ |
| 9 | HFE testing should be considered in patients with unexplained chronic liver disease pre-selected for increased transferrin saturation | 1C | EASL | **AASLD** (A)  Patients with abnormal iron studies should be evaluated as patients with hemochromatosis, even in the absence of symptoms | **1 2 3 4 5 6 7 8 9**  **<----------------------------------------------------------->**  **disagree agree**  Impossible to judge □ |

***Prioritization ‘Screening’***

**What in your opinion are the 3 most important recommendations in order to measure high quality of care for HH patients with respect to screening, with the first recommendation being the most important?**

|  | **Number** | **Recommendation** | **Motivation** |
| --- | --- | --- | --- |
| 1 |  |  |  |
| 2 |  |  |  |
| 3 |  |  |  |

| **2. Diagnosis** | | | | | |
| --- | --- | --- | --- | --- | --- |
| **Performing this recommendation is important for the delivery of high quality care for patients with hereditary haemochromatosis** | | | | | |
|  | **Recommendation** | **LoE** | **Gdln** | **Comments** | **Score** |
| 10 | In a patient with suggestive symptoms, physical findings, or family history, a combination of TS and ferritin should be obtained. If either is abnormal (TS > 45% **OR** ferritin above upper limit of normal), HFE mutation analysis should be performed | 1B | AASLD | **DUTCH**  Research of HFE mutations should be done in all patients with TS > 45% **AND** serum ferritin above normal values of the lab for age and sex, all other causes of elevated iron parameters should be excluded  **EASL**  Patients with suspected iron overload should first receive measurement of fasting transferrin saturation and serum ferritin (1B), and HFE testing should be performed **ONLY** in those with increased transferrin saturation (1A) | **1 2 3 4 5 6 7 8 9**  **<----------------------------------------------------------->**  **disagree agree**  Impossible to judge □ |
| 11 | Patients from liver clinics should be screened for fasting transferrin saturation and serum ferritin | 1C | EASL | **AASLD** (1B)  All patients with evidence of liver disease should be evaluated for hemochromatosis | **1 2 3 4 5 6 7 8 9**  **<----------------------------------------------------------->**  **disagree agree**  Impossible to judge □ |
| 12 | Patients from liver clinics should be offered genetic HFE testing if transferrin saturation is increased | 1B | EASL |  | **1 2 3 4 5 6 7 8 9**  **<----------------------------------------------------------->**  **disagree agree**  Impossible to judge □ |
| 13 | HFE testing for the C282Y and H63D polymorphism should be carried out in all patients with otherwise unexplained increased serum ferritin and transferrin saturation | 1B | EASL |  | **1 2 3 4 5 6 7 8 9**  **<----------------------------------------------------------->**  **disagree agree**  Impossible to judge □ |
| 14 | Diagnosis of HFE hemochromatosis should not be based on C282Y homozygosity alone, but requires evidence of increased iron stores | 1B | EASL |  | **1 2 3 4 5 6 7 8 9**  **<----------------------------------------------------------->**  **disagree agree**  Impossible to judge □ |
| 15 | In C282Y homozygote patients with increased iron stores, liver biopsy is no longer necessary to diagnose hemochromatosis | 1C | EASL |  | **1 2 3 4 5 6 7 8 9**  **<----------------------------------------------------------->**  **disagree agree**  Impossible to judge □ |
| 16 | Diagnostic strategies using serum iron markers should target high-risk groups such as those with family history of HH or those with suspected organ involvement | 1B | AASLD |  | **1 2 3 4 5 6 7 8 9**  **<----------------------------------------------------------->**  **disagree agree**  Impossible to judge □ |

***Prioritization ‘Diagnosis’***

**What in your opinion are the 3 most important recommendations in order to measure high quality of care for HH patients with respect to diagnosis, with the first recommendation being the most important?**

|  | **Number** | **Recommendation** | **Motivation** |
| --- | --- | --- | --- |
| 1 |  |  |  |
| 2 |  |  |  |
| 3 |  |  |  |

| **3. Treatment and management** | | | | | |
| --- | --- | --- | --- | --- | --- |
| **Performing this recommendation is important for the delivery of high quality care for patients with hereditary haemochromatosis** | | | | | |
|  | **Recommendation** | **LoE** | **Gdln** | **Comments** | **Score** |
| **Phlebotomy** | | | | | |
| 17 | Patients with HFE-HC and evidence of excess iron should be treated with phlebotomy | 1C | EASL  DUTCH |  | **1 2 3 4 5 6 7 8 9**  **<----------------------------------------------------------->**  **disagree agree**  Impossible to judge □ |
| 18 | Phlebotomy should be carried out by removing 400-500 ml of blood (200-250mg iron) weekly or every two weeks. | 1C  4 | EASL  DUTCH | **AASLD** (1A)  Patients with hemochromatosis and iron overload should undergo therapeutic phlebotomy **weekly** (as tolerated) | **1 2 3 4 5 6 7 8 9**  **<----------------------------------------------------------->**  **disagree agree**  Impossible to judge □ |
| 19 | Phlebotomy can also be performed in patients with advanced fibrosis or cirrhosis | 2C  2 | EASL  DUTCH | **AASLD** (1A)  Patients with end-organ damage due to iron overload should undergo regular phlebotomy in case of iron overload | **1 2 3 4 5 6 7 8 9**  **<----------------------------------------------------------->**  **disagree agree**  Impossible to judge □ |
| 20 | Adequate hydration before and after treatment, and avoidance of vigorous physical activity for 24h after phlebotomy is recommended | 1C | EASL |  | **1 2 3 4 5 6 7 8 9**  **<----------------------------------------------------------->**  **disagree agree**  Impossible to judge □ |
| 21 | Target level of phlebotomy is a ferritin level of 50-100 µg/L | 1B  4 | AALSD  DUTCH |  | **1 2 3 4 5 6 7 8 9**  **<----------------------------------------------------------->**  **disagree agree**  Impossible to judge □ |
| 22 | In the absence of indicators suggestive of significant liver disease (ALT, AST elevation), C282Y homozygotes with elevated ferritin (but < 1000 µg/L) should proceed to phlebotomy | 1B | AASLD |  | **1 2 3 4 5 6 7 8 9**  **<----------------------------------------------------------->**  **disagree agree**  Impossible to judge □ |

| **Liver biopsy** | | | | | |
| --- | --- | --- | --- | --- | --- |
| 23 | Liver biopsy is recommended to stage the degree of liver disease in **C282Y homozygotes or compound heterozygotes** if liver enzymes (ALT, AST) are elevated **OR** if ferritin is > 1000 µg/L | 1B | AASLD | **EASL** (1C)  Liver biopsy could be offered to **C282Y homozygote** patients with serum ferritin above 1000 µg/L, elevated AST, hepatomegaly, or age over 40 years  **DUTCH** (2)  Liver biopsy should be used to determine the stage of liver damage if serum ferritin > 1000 µg/L | **1 2 3 4 5 6 7 8 9**  **<----------------------------------------------------------->**  **disagree agree**  Impossible to judge □ |
| **Examinations** | | | | | |
| 24 | **Transient elastography** can be helpful for the demonstration of advanced fibrosis and cirrhosis (in HH/HC patients) | 1C | EASL |  | **1 2 3 4 5 6 7 8 9**  **<----------------------------------------------------------->**  **disagree agree**  Impossible to judge □ |
| 25 | MRI can be helpful to (I) identify heterogeneous distribution of iron within the liver, (II) differentiate parenchymal from mesenchymal iron overload, and (III) detect small iron-free neoplastic lesions. |  | EASL | **DUTCH** (2)  MRI is a non-invasive way to do a clinical useful, semi-quantitative iron determination of the liver | **1 2 3 4 5 6 7 8 9**  **<----------------------------------------------------------->**  **disagree agree**  Impossible to judge □ |
| **General** | | | | | |
| 26 | C282Y homozygotes without evidence of iron overload can be monitored **annually** and treatment instituted when the ferritin rises above normal. | 2C  TEXT | EASL  AASLD | **DUTCH**  If no deviations, new monitoring every three years | **1 2 3 4 5 6 7 8 9**  **<----------------------------------------------------------->**  **disagree agree**  Impossible to judge □ |
| 27 | To minimize the risk of additional complications, patients with HFE-HC can be immunized against hepatitis A and B while iron overloaded | 2C | EASL |  | **1 2 3 4 5 6 7 8 9**  **<----------------------------------------------------------->**  **disagree agree**  Impossible to judge □ |
| 28 | Cirrhotic HFE-HC patients should be immunized against influenza yearly |  | Article 1 |  | **1 2 3 4 5 6 7 8 9**  **<----------------------------------------------------------->**  **disagree agree**  Impossible to judge □ |
| 29 | Cirrhotic HFE-HC patients should be immunized against pneumococci every 5 years |  | Article 1 |  | **1 2 3 4 5 6 7 8 9**  **<----------------------------------------------------------->**  **disagree agree**  Impossible to judge □ |
| 30 | Before initiation of phlebotomy, patients with HFE-HC should be assessed for complications including diabetes mellitus, joint disease, endocrine deficiency (hypothyroidism), cardiac disease, porphyria cutanea tarda, and osteoporosis | 1C | EASL |  | **1 2 3 4 5 6 7 8 9**  **<----------------------------------------------------------->**  **disagree agree**  Impossible to judge □ |
| 31 | Complications of HFE-HC (liver cirrhosis, diabetes, arthropathy, hypogonadism, PCT) should be managed regardless whether or not HC is the underlying cause and whether there is symptomatic relief or improvement during phlebotomy | 1C | EASL |  | **1 2 3 4 5 6 7 8 9**  **<----------------------------------------------------------->**  **disagree agree**  Impossible to judge □ |
| 32 | HFE-HC patients with cirrhosis should be screened for focal liver lesions, using ultrasound examination and serum alpha fetoprotein measurement every 6 months. | TEXT | EASL  DUTCH |  | **1 2 3 4 5 6 7 8 9**  **<----------------------------------------------------------->**  **disagree agree**  Impossible to judge □ |
| 33 | Fasting glycemia and/or HbA1c should be monitored regularly to detect diabetes mellitus | TEXT | EASL |  | **1 2 3 4 5 6 7 8 9**  **<----------------------------------------------------------->**  **disagree agree**  Impossible to judge □ |
| 34 | Physical and radiological evaluation is necessary to evaluate possible arthralgia and arthritis | TEXT | EASL |  | **1 2 3 4 5 6 7 8 9**  **<----------------------------------------------------------->**  **disagree agree**  Impossible to judge □ |
| 35 | In case of any cardiac symptoms, an electrocardiogram, echocardiography, and 24h ambulatory ECG monitoring should be performed | TEXT | EASL |  | **1 2 3 4 5 6 7 8 9**  **<----------------------------------------------------------->**  **disagree agree**  Impossible to judge □ |
| 36 | Thyroid function tests and serum testosterone levels should be monitored regularly? Yearly? | TEXT | EASL |  | **1 2 3 4 5 6 7 8 9**  **<----------------------------------------------------------->**  **disagree agree**  Impossible to judge □ |
| 37 | Patients with HFE-HC are at risk of osteoporosis, and should undergo a DEXA scan and receive appropriate routine advice or treatment for osteoporosis if diagnosed | TEXT | EASL |  | **1 2 3 4 5 6 7 8 9**  **<----------------------------------------------------------->**  **disagree agree**  Impossible to judge □ |
| **Diet – lifestyle** | | | | | |
| 38 | During treatment for HH, dietary adjustments are unnecessary. Vitamin C supplements and iron supplements should be avoided | 1C  4  TEXT | AASLD  DUTCH  EASL |  | **1 2 3 4 5 6 7 8 9**  **<----------------------------------------------------------->**  **disagree agree**  Impossible to judge □ |
| 39 | Patients with elevated iron parameters during depletion phase should avoid the intake of alcohol | 3 | DUTCH | **EASL** (TEXT)  Excess alcohol ingestion leads to increased hepatic damage in HFE-HC | **1 2 3 4 5 6 7 8 9**  **<----------------------------------------------------------->**  **disagree agree**  Impossible to judge □ |
| 40 | HFE-HC patients should avoid the intake of a lot of red meat | 4 | DUTCH |  | **1 2 3 4 5 6 7 8 9**  **<----------------------------------------------------------->**  **disagree agree**  Impossible to judge □ |
| 41 | HFE-HC patients should avoid drinking black tea during nutrition | 4 | DUTCH |  | **1 2 3 4 5 6 7 8 9**  **<----------------------------------------------------------->**  **disagree agree**  Impossible to judge □ |

***Prioritization ‘treatment/management’***

**What in your opinion are the 3 most important recommendations in order to measure high quality of care for HH patients with respect to treatment and management, with the first recommendation being the most important?**

|  | **Number** | **Recommendation** | **Motivation** |
| --- | --- | --- | --- |
| 1 |  |  |  |
| 2 |  |  |  |
| 3 |  |  |  |

**Additional recommendations/modifications**

|  | **Number** | **Additional recommendation/modification** |
| --- | --- | --- |
| 1 |  |  |
| 2 |  |  |
| 3 |  |  |
| 4 |  |  |
| 5 |  |  |
| 6 |  |  |
| 7 |  |  |
| 8 |  |  |
| 9 |  |  |
| 10 |  |  |

**Attachment I: Process of indicator development**

**Selection of recommendations from international guidelines**

STEP 1

**Existing information on immunization of cirrhotic patients**

**Written questionnaire (first round)**

- Screening
- Diagnosis
- Treatment/management

**Transformation of recommendations into quality indicators**

**Critical appraisal: determination of core set of recommendations**

- Measurability
- Improvement potential

Final approval by expert team

**Consensus meeting (second round) 🡪 19/12/2013**

- Mean score
- Agreement
- prioritization

**Processing results of first round**

- Mean score
- Agreement
- prioritization

**Additional/modified items**

STEP 2

STEP 3

STEP 4

STEP 5

STEP 6

**Attachment II: References**

**EASL** European Association For The Study Of The Liver. EASL clinical practice guidelines for HFE hemochromatosis. J Hepatol 2010;53:3-22.

**AASLD** Bacon BR, Adams PC, Kowdley KV, et al. Diagnosis and management of hemochromatosis: 2011 Practice Guideline by the American Association for the Study of Liver Diseases. Hepatology 2011;54:328-43.

**DUTCH** Richtlijn Hereditaire Hemochromatose. Diagnostiek en behandeling van hereditaire hemochromatose. 2007. Date accessed on June 2th 2013 via <http://www.internisten.nl/uploads/AL/FJ/ALFJChidQ2OgF7uax8yh1w/richtlijn_2007_Hemochromatose.pdf>

**Article 1** Loulergue P, Pol S, Mallet V, Sogni P, Launay O. Why actively promote vaccination in patients with cirrhosis? J Clin Virol 2009;46:206-9.

**Attachment III: Level of evidence**

**EASL**

**
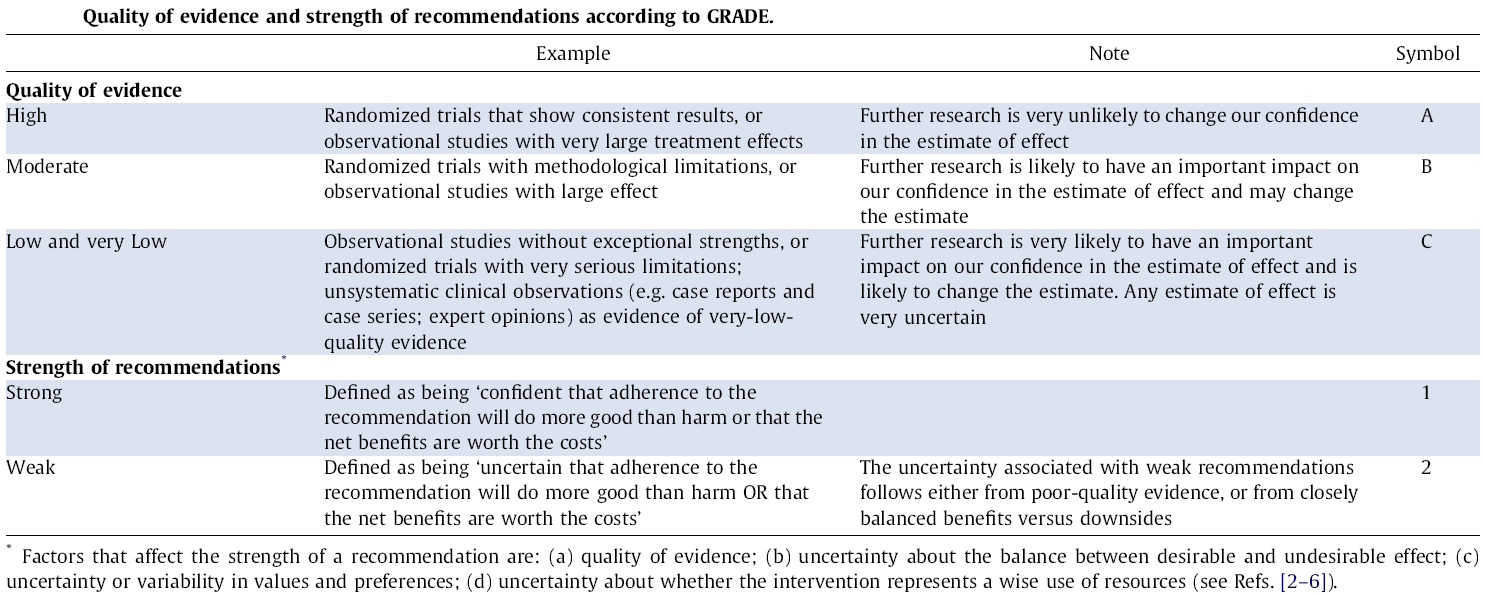
**

**AASLD**

**
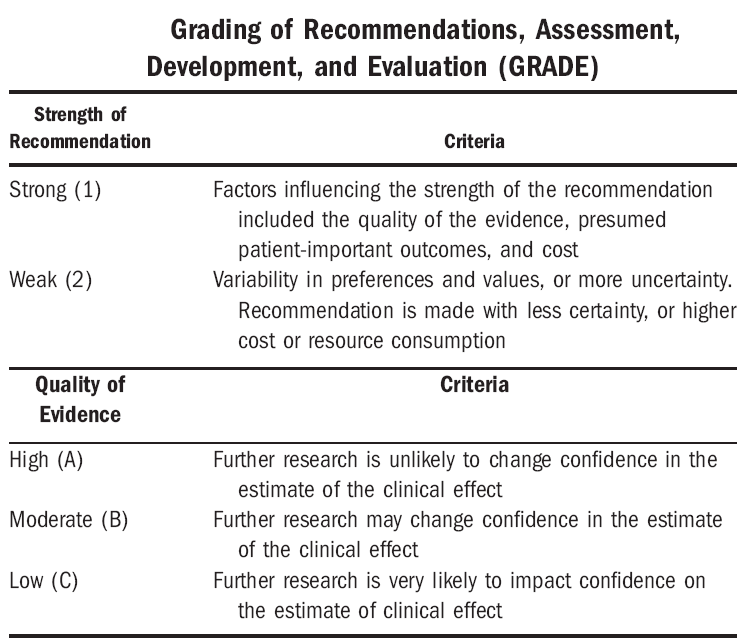
**

**DUTCH**

**Strength of recommendation according to CBO**

| Level of evidence of the conclusions | |
| --- | --- |
| Level 1 | **Based on 1 systematic review or at least 2 independent randomized double-blind controlled trials** |
| Level 2 | **Based on at least 2 independent trials not of ‘level 1’ quality** |
| Level 3 | **Based on one trial of ‘level 1’ quality or lower** |
| Level 4 | **Based on expert opinion** |
