# Supplementary material for: Key-interventions derived from three evidence based guidelines for management and follow-up of patients with HFE haemochromatosis
Source: BMC Health Serv Res. 2016 Oct 13;16:573. doi: 10.1186/s12913-016-1835-2 (PMC5062877; doi:10.1186/s12913-016-1835-2)
Supplement: Additional file 2: Appendix B. — Expert overview. (DOCX 14 kb) [file 12913_2016_1835_MOESM2_ESM.docx]

**Experts**

**Hepatology**

Prof. Dr. David Cassiman – University Hospital Gasthuisberg, Leuven

Prof. Dr. Hans Van Vlierberghe – University Hospital Ghent

Dr. Rudy Harlet – Academic Hospital Turnhout

**Rheumatology**

Prof. Dr. Rene Westhovens - University Hospital Gasthuisberg, Leuven

Prof. Dr. Patrick Verschueren - University Hospital Gasthuisberg, Leuven

**Cardiology**

Prof. dr. Johan Van Cleemput - University Hospital Gasthuisberg, Leuven

Dr. Walter Droogne - University Hospital Gasthuisberg, Leuven

**Endocrinology**

Prof. Dr. Chantal Mathieu - University Hospital Gasthuisberg, Leuven (only info about endocrinology)

**Hematology**

Dr. Vincent Maertens – Imelda Hospital Bonheiden

Dr. Koen Theunissen – Jessa Hospital Hasselt

**General Practitioners**

Prof. Dr. Neree Claes – University of Hasselt

Prof. Dr. Patrik Vankrunkelsven – University of Leuven

Dr. Hannelore Van Droogenbroeck – private practice

**Nurses**

Sabien Severi – University Hospital Gasthuisberg, Leuven

Ilse Scherens – University Hospital Gasthuisberg, Leuven

Sandra Rottiers – University Hospital Gasthuisberg, Leuven
