# Supplementary material for: Key-interventions derived from three evidence based guidelines for management and follow-up of patients with HFE haemochromatosis
Source: BMC Health Serv Res. 2016 Oct 13;16:573. doi: 10.1186/s12913-016-1835-2 (PMC5062877; doi:10.1186/s12913-016-1835-2)
Supplement: Additional file 3: Appendix C. — Overview scores written questionnaire. (DOCX 64 kb) [file 12913_2016_1835_MOESM3_ESM.docx]

High

Low

middle

| Nr | Description | Total | 1 | 2 | 3 | 4 | 5 | 6 | 7 | 8 | 9 | % highest | Median | Top1 | Top2 | Top3 | Point | Max | % top3 | Conclusion |
| --- | --- | --- | --- | --- | --- | --- | --- | --- | --- | --- | --- | --- | --- | --- | --- | --- | --- | --- | --- | --- |
| Topic 1: SCREENING | | | | | | | | | | | | | | | | | | | | |
| 1 | Population screening should not be done | 14 | 0 | 0 | 0 | 0 | 1 | 2 | 0 | 8 | 3 | 79 | 8 | 0 | 2 | 3 | 7 | 42 | 17% | Discussion |
| 2 | Genetic testing of first-degree relatives should be considered | 15 | 0 | 0 | 0 | 0 | 0 | 1 | 0 | 4 | 10 | 93 | 9 | 13 | 0 | 1 | 40 | 45 | 89% | Selection |
| 3 | HFE testing must be considered in patients with porphyria cutanea tarda | 11 | 0 | 0 | 0 | 0 | 1 | 1 | 2 | 4 | 3 | 82 | 8 | 0 | 2 | 1 | 5 | 33 | 15% | Discussion |
| 4 | HFE testing must be considered in patients with well-defined chondrocalcinosis | 12 | 0 | 1 | 0 | 1 | 3 | 3 | 3 | 1 | 0 | 33 | 6 | 0 | 0 | 0 | 0 | 36 | 0% | No selection |
| 5 | HFE testing must be considered in patients with hepatocellular carcinoma | 12 | 0 | 2 | 0 | 1 | 2 | 1 | 4 | 0 | 2 | 50 | 6.5 | 0 | 1 | 1 | 3 | 36 | 8% | No selection |
| 6 | HFE testing must be considered in patients with type 1 diabetes | 12 | 0 | 1 | 1 | 0 | 2 | 2 | 3 | 1 | 2 | 50 | 6.5 | 0 | 0 | 4 | 4 | 36 | 11% | No selection |
| 7 | HFE testing should not be done in patients with unexplained arthritis or arthralgia | 13 | 1 | 1 | 0 | 1 | 0 | 2 | 1 | 3 | 4 | 62 | 8 | 1 | 1 | 0 | 5 | 39 | 13% | No selection |
| 8 | HFE testing should not be done in patients with type 2 diabetes | 11 | 0 | 0 | 1 | 1 | 1 | 0 | 1 | 3 | 4 | 73 | 8 | 0 | 0 | 1 | 1 | 33 | 3% | Discussion |
| 9 | HFE testing should be considered in patients with unexplained chronic liver disease pre-selected for increased transferrin saturation | 14 | 0 | 0 | 0 | 0 | 1 | 0 | 0 | 6 | 7 | 93 | 8.5 | 1 | 9 | 1 | 22 | 42 | 52% | Selection |
| Topic 2: DIAGNOSIS | | | | | | | | | | | | | | | | | | | | |
| 10 | In a patient with suggestive symptoms, physical findings, or family history, a combination of TS and ferritin should be obtained. If either is abnormal (TS > 45% OR ferritin above upper limit of normal), HFE mutation analysis should be performed | 14 | 0 | 0 | 0 | 0 | 0 | 1 | 1 | 4 | 8 | 93 | 9 | 8 | 1 | 1 | 27 | 42 | 64% | Selection |
| 11 | Patients from liver clinics should be screened for fasting transferrin saturation and serum ferritin | 13 | 0 | 0 | 0 | 0 | 1 | 1 | 1 | 4 | 6 | 85 | 8 | 2 | 7 | 0 | 20 | 39 | 51% | Selection |
| 12 | Patients from liver clinics should be offered genetic HFE testing if transferrin saturation is increased | 14 | 0 | 0 | 0 | 0 | 2 | 0 | 1 | 3 | 8 | 86 | 9 | 0 | 0 | 6 | 6 | 42 | 14% | Discussion |
| 13 | HFE testing for the C282Y and H63D polymorphism should be carried out in all patients with otherwise unexplained increased serum ferritin and transferrin saturation | 12 | 0 | 0 | 0 | 0 | 0 | 1 | 1 | 4 | 6 | 92 | 8.5 | 1 | 3 | 1 | 10 | 36 | 28% | Selection |
| 14 | Diagnosis of HFE hemochromatosis should not be based on C282Y homozygosity alone, but requires evidence of increased iron stores | 11 | 0 | 0 | 0 | 0 | 0 | 1 | 6 | 3 | 1 | 91 | 7 | 0 | 1 | 1 | 3 | 33 | 9% | Discussion |
| 15 | In C282Y homozygote patients with increased iron stores, liver biopsy is no longer necessary to diagnose hemochromatosis | 13 | 0 | 0 | 0 | 1 | 0 | 0 | 4 | 3 | 5 | 92 | 8 | 1 | 1 | 2 | 7 | 39 | 18% | Discussion |
| 16 | Diagnostic strategies using serum iron markers should target high-risk groups such as those with family history of HH or those with suspected organ involvement | 13 | 0 | 0 | 0 | 0 | 1 | 0 | 3 | 5 | 4 | 92 | 8 | 1 | 0 | 1 | 4 | 39 | 10% | Discussion |
| Topic 3: TREATMENT/MANAGEMENT | | | | | | | | | | | | | | | | | | | | |
| Phlebotomy | | | | | | | | | | | | | | | | | | | | |
| 17 | Patients with HFE-HC and evidence of excess iron should be treated with phlebotomy | 14 | 0 | 0 | 0 | 0 | 1 | 1 | 1 | 4 | 7 | 86 | 8.5 | 9 | 2 | 0 | 31 | 42 | 74% | Selection |
| 18 | Phlebotomy should be carried out by removing 400-500 ml of blood (200-250mg iron) weekly or every two weeks. | 13 | 0 | 0 | 0 | 0 | 2 | 0 | 3 | 5 | 3 | 85 | 8 | 1 | 2 | 1 | 8 | 39 | 21% | Selection |
| 19 | Phlebotomy can also be performed in patients with advanced fibrosis or cirrhosis | 10 | 0 | 0 | 0 | 0 | 1 | 3 | 1 | 2 | 3 | 60 | 7.5 | 0 | 0 | 0 | 0 | 30 | 0% | No selection |
| 20 | Adequate hydration before and after treatment, and avoidance of vigorous physical activity for 24h after phlebotomy is recommended | 14 | 0 | 0 | 0 | 0 | 1 | 2 | 1 | 6 | 4 | 79 | 8 | 0 | 1 | 0 | 2 | 42 | 5% | Discussion |
| 21 | Target level of phlebotomy is a ferritin level of 50-100 µg/L | 14 | 0 | 0 | 0 | 0 | 0 | 0 | 6 | 7 | 1 | 100 | 8 | 0 | 3 | 3 | 9 | 42 | 21% | Selection |
| 22 | In the absence of indicators suggestive of significant liver disease (ALT, AST elevation), C282Y homozygotes with elevated ferritin (but < 1000 µg/L) should proceed to phlebotomy | 12 | 0 | 1 | 0 | 0 | 0 | 1 | 1 | 6 | 3 | 83 | 8 | 0 | 0 | 0 | 0 | 36 | 0% | Discussion |
| Liver biopsy | | | | | | | | | | | | | | | | | | | | |
| 23 | Liver biopsy is recommended to stage the degree of liver disease in C282Y homozygotes or compound heterozygotes if liver enzymes (ALT, AST) are elevated OR if ferritin is > 1000 µg/L | 12 | 0 | 1 | 2 | 0 | 1 | 2 | 3 | 3 | 0 | 50 | 6.5 | 0 | 0 | 0 | 0 | 36 | 0% | No selection |
| Examinations | | | | | | | | | | | | | | | | | | | | |
| 24 | Transient elastography can be helpful for the demonstration of advanced fibrosis and cirrhosis (in HH/HC patients) | 10 | 0 | 0 | 0 | 0 | 3 | 0 | 2 | 5 | 0 | 70 | 7.5 | 0 | 0 | 1 | 1 | 30 | 3% | Discussion |
| 25 | MRI can be helpful to (I) identify heterogeneous distribution of iron within the liver, (II) differentiate parenchymal from mesenchymal iron overload, and (III) detect small iron-free neoplastic lesions. | 12 | 0 | 0 | 0 | 0 | 1 | 1 | 4 | 4 | 2 | 83 | 7.5 | 0 | 0 | 0 | 0 | 36 | 0% | Discussion |
| General | | | | | | | | | | | | | | | | | | | | |
| 26 | C282Y homozygotes without evidence of iron overload can be monitored annually and treatment instituted when the ferritin rises above normal. | 14 | 0 | 0 | 0 | 1 | 3 | 2 | 3 | 4 | 1 | 57 | 7 | 0 | 0 | 1 | 1 | 42 | 2% | No selection |
| 27 | To minimize the risk of additional complications, patients with HFE-HC can be immunized against hepatitis A and B while iron overloaded | 13 | 0 | 0 | 0 | 0 | 1 | 1 | 4 | 4 | 3 | 85 | 8 | 0 | 3 | 0 | 6 | 39 | 15% | Discussion |
| 28 | Cirrhotic HFE-HC patients should be immunized against influenza yearly | 13 | 0 | 0 | 0 | 0 | 1 | 0 | 1 | 6 | 5 | 92 | 8 | 0 | 0 | 2 | 2 | 39 | 5% | Discussion |
| 29 | Cirrhotic HFE-HC patients should be immunized against pneumococci every 5 years | 12 | 0 | 1 | 0 | 0 | 0 | 0 | 1 | 5 | 5 | 92 | 8 | 0 | 0 | 0 | 0 | 36 | 0% | Discussion |
| 30 | Before initiation of phlebotomy, patients with HFE-HC should be assessed for complications including diabetes mellitus, joint disease, endocrine deficiency (hypothyroidism), cardiac disease, porphyria cutanea tarda, and osteoporosis | 15 | 0 | 1 | 0 | 0 | 1 | 1 | 3 | 6 | 3 | 80 | 8 | 1 | 1 | 0 | 5 | 45 | 11% | Discussion |
| 31 | Complications of HFE-HC (liver cirrhosis, diabetes, arthropathy, hypogonadism, PCT) should be managed regardless whether or not HC is the underlying cause and whether there is symptomatic relief or improvement during phlebotomy | 13 | 0 | 0 | 0 | 0 | 0 | 0 | 2 | 5 | 6 | 100 | 8 | 1 | 1 | 2 | 7 | 39 | 18% | Discussion |
| 32 | HFE-HC patients with cirrhosis should be screened for focal liver lesions, using ultrasound examination and serum alpha fetoprotein measurement every 6 months. | 13 | 0 | 0 | 0 | 0 | 1 | 1 | 0 | 7 | 4 | 85 | 8 | 0 | 0 | 1 | 1 | 39 | 3% | Discussion |
| 33 | Fasting glycemia and/or HbA1c should be monitored regularly to detect diabetes mellitus | 14 | 0 | 0 | 0 | 0 | 1 | 2 | 2 | 3 | 6 | 79 | 8 | 0 | 0 | 0 | 0 | 42 | 0% | Discussion |
| 34 | Physical and radiological evaluation is necessary to evaluate possible arthralgia and arthritis | 13 | 0 | 0 | 2 | 0 | 1 | 4 | 3 | 3 | 0 | 46 | 6 | 0 | 0 | 0 | 0 | 39 | 0% | No selection |
| 35 | In case of any cardiac symptoms, an electrocardiogram, echocardiography, and 24h ambulatory ECG monitoring should be performed | 14 | 0 | 0 | 0 | 0 | 0 | 2 | 3 | 6 | 3 | 86 | 8 | 1 | 0 | 0 | 3 | 42 | 7% | Discussion |
| 36 | Thyroid function tests and serum testosterone levels should be monitored regularly? Yearly? | 12 | 0 | 0 | 0 | 0 | 1 | 1 | 4 | 5 | 1 | 83 | 7.5 | 0 | 0 | 0 | 0 | 36 | 0% | Discussion |
| 37 | Patients with HFE-HC are at risk of osteoporosis, and should undergo a DEXA scan and receive appropriate routine advice or treatment for osteoporosis if diagnosed | 12 | 0 | 0 | 0 | 0 | 3 | 0 | 4 | 5 | 0 | 75 | 7 | 0 | 0 | 0 | 0 | 36 | 0% | Discussion |
| Diet – lifestyle | | | | | | | | | | | | | | | | | | | | |
| 38 | During treatment for HH, dietary adjustments are unnecessary. Vitamin C supplements and iron supplements should be avoided | 15 | 0 | 0 | 0 | 0 | 2 | 0 | 2 | 5 | 6 | 87 | 8 | 1 | 0 | 0 | 3 | 45 | 7% | Discussion |
| 39 | Patients with elevated iron parameters during depletion phase should avoid the intake of alcohol | 14 | 0 | 0 | 0 | 1 | 0 | 3 | 3 | 4 | 3 | 71 | 7.5 | 1 | 1 | 1 | 6 | 42 | 14% | Discussion |
| 40 | HFE-HC patients should avoid the intake of a lot of red meat | 12 | 1 | 1 | 1 | 1 | 2 | 1 | 2 | 3 | 0 | 42 | 5.5 | 0 | 1 | 0 | 2 | 36 | 6% | No selection |
| 41 | HFE-HC patients should avoid drinking black tea during nutrition | 13 | 2 | 1 | 0 | 0 | 8 | 0 | 0 | 0 | 0 | 0 | 5 | 0 | 0 | 1 | 1 | 39 | 3% | No selection |

**Additional recommendations/modifications**

| Nr | Recom. Nr | Modification/additional |
| --- | --- | --- |
| 1 | 1 | Although some data exist, that screening of all men between 25-35 years of age (screening by transferrin saturation) could be effective (Adam et al.) |
| 2 | 3 | HFE testing must be considered in patients with well-defined chondrocalcinosis in case of an otherwise unexplained increase of ferritin and transferrin saturation |
| 3 | 5 | In case no other cause of underlying liver disease is present |
| 4 | 7 | HFE testing should not be done in patients with unexplained arthritis or arthralgia unless there is an unexplained increase of serum ferritin and transferrin saturation |
| 5 | 10 | Agree with the EASL |
| 6 | 14 | This is impossible to judge, since it depends on the definition of HFE hemochromatosis (genetic disease or tissue iron increase) |
| 7 | 18 | What is excess iron: elevated ferritin? Tissue iron? There are some data that when ferritin is not above 1000 ng/ml, one can wait with phlebotomies. |
| 8 | 24 | Agreement with the EASL recommendation |
| 9 | 35 | History and physical examination are necessary to evaluate possible involvement in the joints and in case of doubt radiological evaluation |
| 10 | 36 | Measure also high sensitive troponin and natriuretic peptides. Quid cMR?? |
| 11 | 37 | Thyroid function tests and serum testosterone levels should be monitored **yearly** |
| 12 | 38 | Patients with HFE-HC are at risk of osteoporosis and should undergo a DEXA-scan and FRAX analysis to evaluate their fracture risk. They should receive appropriate advice and/or treatment for osteoporosis if necessary. |
| 13 | 40 | Agree to EASL |
| 14 |  | Should patients with an otherwise unexplained dilated cardiomyopathy be screened for hemochromatosis and how: TS, other, …? Under the heading “diagnostic tests: recommendations” the most recent ACC/AHA guidelines for hearth failure (Circulation 2013;62:e147-239) suggest: screening for hemochromatosis or HIV is reasonable in selected patients who present with hearth failure (category IIA level c) |
| 15 |  | Screening for hemochromatosis should be done in all patients with diabetes with atypical presentation |
| 16 |  | Screening for HC may be done in people with Type 1 diabetes without autoantibodies against the beta-cell. |
| 17 |  | Not starting with phlebotomies: age related (upper limit)? |
