# Supplementary material for: Key-interventions derived from three evidence based guidelines for management and follow-up of patients with HFE haemochromatosis
Source: BMC Health Serv Res. 2016 Oct 13;16:573. doi: 10.1186/s12913-016-1835-2 (PMC5062877; doi:10.1186/s12913-016-1835-2)
Supplement: Additional file 4: Appendix D. — Selected recommendations. (DOCX 18 kb) [file 12913_2016_1835_MOESM4_ESM.docx]

**Selection and adaptation of recommendations after consensus meeting**

After the initial written scoring form, we ended up with 8 recommendations with a high, 10 recommendations with a low and 23 recommendations with an uncertain potential to deliver high quality of care in patients with hereditary haemochromatosis (HH). Some recommendations were adapted and three recommendations were added: 1) Screening for hemochromatosis should be done in all patients with diabetes with atypical presentation. 2) Screening for hemochromatosis may be done in people with type 1 diabetes without antibodies against the beta-cell. 3) Patients should not start with phlebotomies after a certain age. None of them were added to the final list.

Considering the results of the written questionnaire, the recommendations were discussed during the consensus meeting. 28 recommendations were selected as having a high potential for delivering high quality of care in patients with HH. 13 recommendations were excluded.

| Nr | Selection of recommendation after consensus meeting | Comments? |
| --- | --- | --- |
| Screening | | |
| 1 | Genetic testing of first-degree relatives should be considered | If a patient is diagnosed with HH, first-degree relatives should be screened. |
| 2 | HFE testing must be considered in patients with well-defined chondrocalcinosis in case of an otherwise unexplained increase of ferritin and transferrin saturation |  |
| 3 | HFE testing must be considered in patients with type 1 diabetes in case of abnormal iron parameters |  |
| 4 | HFE testing should not be done in patients with unexplained osteoarthritis |  |
| 5 | HFE testing should be considered in patients with type 2 diabetes in case of abnormal iron parameters |  |
| 6 | HFE testing should be considered in patients with unexplained chronic liver disease pre-selected for increased transferrin saturation |  |
| Diagnosis | | |
| 7 | In a patient with suggestive symptoms, physical findings, or family history, a combination of TS and ferritin should be obtained. If both are abnormal (TS > 45% AND ferritin above upper limit of normal), HFE mutation analysis should be performed |  |
| 8 | Patients from liver clinics should be screened for transferrin saturation and serum ferritin |  |
| 9 | Patients from liver clinics should be offered genetic HFE testing if transferrin saturation and ferritin is increased |  |
| 10 | HFE testing for the C282Y and H63D polymorphism should be carried out in all patients with otherwise unexplained increased serum ferritin and transferrin saturation |  |
| 11 | In C282Y homozygote patients with increased iron stores, liver biopsy is no longer necessary to diagnose hemochromatosis |  |
| Treatment/management | | |
| Phlebotomy | | |
| 12 | Patients with HFE-HC and evidence of excess iron should be treated with phlebotomy | If patients with HH have abnormal iron parameters, they should be treated with phlebotomy |
| 13 | Phlebotomy should start by removing 400-500 ml of blood (200-250mg iron) weekly or every two weeks | If patients with HH start phlebotomy, they should start (bi) weekly by removing 400-500 ml of blood |
| 14 | Phlebotomy can also be performed in patients with advanced fibrosis or cirrhosis | If patients with HH have advanced fibrosis or cirrhosis, phlebotomy can also performed |
| 15 | Adequate hydration before and after treatment, and avoidance of vigorous physical activity for 24h after phlebotomy is recommended | If patients with HH have phlebotomies, they should be advised to have adequate hydration before and after treatment. They should be advised to avoid vigorous physical activity for 24h |
| 16 | Target level of phlebotomy is a ferritin level of 50-100 µg/L | If patients with HH have phlebotomies, the ferritin target level is between 50-100µg/L |
| 17 | In the absence of indicators suggestive of significant liver disease (ALT, AST elevation), C282Y homozygotes with elevated ferritin (but < 1000 µg/L) should proceed to phlebotomy | If patients with HH have no indicators for significant liver disease (ALT, AST elevation), C282Y homozygotes with elevated ferritin (but < 1000 µg/L) should proceed to phlebotomy |
| Examinations | | |
| 18 | Examinations (transient elastography, Biopsy) can be helpful for the demonstration of advanced fibrosis and cirrhosis (in HH/HC patients) |  |
| General | | |
| 19 | C282Y homozygotes without evidence of iron overload should be monitored annually and treatment instituted when the ferritin rises above normal | Patients with HH (C282Y homozygotes) without evidence of iron overload should be monitored annually and treated when the ferritin rises above normal |
| 20 | To minimize the risk of additional complications, patients with HFE-HC should be immunized against hepatitis A and B while iron overloaded | Patients with HH should be immunized against hepatitis A and B |
| 21 | Cirrhotic HFE-HC patients should be immunized against influenza yearly | If patients with HH have cirrhosis, the patient should be immunized against influenza yearly |
| 22 | Cirrhotic HFE-HC patients should be immunized against pneumococci every 5 years | If patients with HH have cirrhosis, the patient should be immunized against pneumococci every 5 years |
| 23 | Patients with HFE-HC should be assessed for complications including diabetes mellitus, joint disease, endocrine deficiency (hypothyroidism), cardiac disease, porphyria cutanea tarda, and osteoporosis | Patients with HH should be assessed for complications including diabetes mellitus, joint disease, endocrine deficiency (hypothyroidism), cardiac disease, porphyria cutanea tarda and osteoporosis |
| 24 | Complications of HFE-HC (liver cirrhosis, diabetes, arthropathy, hypogonadism, PCT) should be managed | Patients with HH should be managed for complications (liver cirrhosis, diabetes mellitus, arthropathy, hypogonadism, PCT) |
| 25 | HFE-HC patients with cirrhosis should be screened for focal liver lesions, using ultrasound examination and serum alpha fetoprotein measurement every 6 months | If patients with HH has cirrhosis, they should be screened for focal liver lesions every six months (ultrasound and serum alpha fetoprotein) |
| 26 | Fasting glycemia and/or HbA1c should be monitored yearly to detect diabetes mellitus | In patients with HH, yearly fasting glycemia and/or HbA1c should be measured to detect diabetes mellitus |
| 27 | Physical and radiological evaluation is necessary to evaluate possible osteoarthritis | If patients with HH has possible osteoarthritis, physical and radiological evaluation need to be done |
| Diet/lifestyle | | |
| 28 | Patients with elevated iron parameters during depletion phase should be advised to avoid the intake of alcohol | If patients with HH are at depletion phase, they should avoid the intake of alcohol. |

If patients with HH have abnormal iron parameters, they should start phlebotomy (bi) weekly by removing 400-500 ml of blood.

| If patients with HH have abnormal iron parameters, they should be treated with phlebotomy |
| --- |
| If patients with HH start phlebotomy, they should start (bi) weekly by removing 400-500 ml of blood |

If patients with HH have cirrhosis, the patient should be immunized against influenza yearly and pneumococci every 5 years.

| If patients with HH have cirrhosis, the patient should be immunized against influenza yearly |
| --- |
| If patients with HH have cirrhosis, the patient should be immunized against pneumococci every 5 years |
